# Inhibition of Pendrin by a small molecule reduces Lipopolysaccharide-induced acute Lung Injury

**DOI:** 10.7150/thno.46417

**Published:** 2020-08-07

**Authors:** Eun Hye Lee, Mi Hwa Shin, Mia Gi, Jinhong Park, Doona Song, Young-Min Hyun, Ji-Hwan Ryu, Je Kyung Seong, Yoon Jeon, Gyoonhee Han, Wan Namkung, Moo Suk Park, Jae Young Choi

**Affiliations:** 1 Division of Pulmonology, Allergy and Critical Care Medicine, Department of Internal Medicine, Yongin Severance Hospital, Yonsei University College of Medicine, Yongin-si, Gyeonggi-do, Republic of Korea.; 2 Division of Pulmonology, Department of Internal Medicine, Institute of Chest Diseases, Severance Hospital, Yonsei University College of Medicine, Seoul, South Korea.; 3 Department of Otorhinolaryngology, Yonsei University College of Medicine, Seoul, South Korea.; 4 The Airway Mucus Institute, Yonsei University College of Medicine, Seoul, South Korea.; 5 College of Pharmacy, Yonsei Institute of Pharmaceutical Sciences, Yonsei University, Incheon, South Korea.; 6 Department of Integrated OMICS for Biomedical Science, Yonsei University, Seoul, South Korea.; 7 Translational Research Center for Protein Function Control, Department of Biotechnology, Yonsei University, Seoul, South Korea.; 8 Department of Anatomy, Yonsei University College of Medicine, Seoul, South Korea.; 9 Severance Biomedical Science Institute, Yonsei University College of Medicine.; 10 Brain Korea 21 PLUS Project for Medical Science, Yonsei University College of Medicine.; 11 Laboratory of Developmental Biology and Genomics, BK21 Plus Program for Advanced Veterinary Science and Research Institute for Veterinary Science, College of Veterinary Medicine, and Korea Mouse Phenotyping Center, Seoul National University, Seoul, South Korea.; 12 Interdisciplinary Program for Bioinformatics, Seoul National University, Seoul, South Korea.; 13 Research Institute, National Cancer Center, South Korea.

**Keywords:** pendrin, inhibitor, SLC26A4, ALI, ARDS

## Abstract

**Rationale:** Pendrin is encoded by *SLC26A4* and its mutation leads to congenital hearing loss. Additionally, pendrin is up-regulated in inflammatory airway diseases such as chronic obstructive pulmonary disease, allergic rhinitis, and asthma. In this study, the effects of a novel pendrin inhibitor, YS-01, were investigated in an LPS-induced acute lung injury (ALI) mice model, and the mechanism underlying the effect of YS-01 was examined.

**Methods:** Lipopolysaccharide (LPS, 10 mg/kg) was intranasally instilled in wild type (WT) and pendrin-null mice. YS-01 (10 mg/kg) was administered intra-peritoneally before or after LPS inhalation. Lung injury parameters were assessed in the lung tissue and bronchoalveolar lavage fluid (BALF). Pendrin levels in the BALF of 41 patients with acute respiratory distress syndrome (ARDS) due to pneumonia and 25 control (solitary pulmonary nodule) patients were also measured.

**Results:** LPS instillation induced lung injury in WT mice but not in pendrin-null mice. Pendrin expression was increased by LPS stimulation both *in vitro* and *in vivo*. YS-01 treatment dramatically attenuated lung injury and reduced BALF cell counts and protein concentration after LPS instillation in WT mice. Proinflammatory cytokines and NF*-κ*B activation were suppressed by YS-01 treatment in LPS-induced ALI mice. In BALF of patients whose ARDS was caused by pneumonia, pendrin expression was up-regulated compared to that in controls (mean, 24.86 vs. 6.83 ng/mL, *P <* 0.001).

**Conclusions:** A novel pendrin inhibitor, YS-01, suppressed lung injury in LPS-induced ALI mice and our data provide a new strategy for the treatment of inflammatory airway diseases including sepsis-induced ALI.

## Introduction

Acute lung injury (ALI), a common and severe pulmonary complication in critical illness, affects approximately 10 to 15% of patients hospitalized in the intensive care unit (ICU) 1. Acute respiratory distress syndrome (ARDS), the most severe form of ALI, has a mortality rate of approximately 40%, despite modern ICU care 2,3. Despite decades of research, treatment options for ARDS are limited and supportive care with mechanical ventilation remains the mainstay of ARDS management 4. ARDS/ALI are characterized by the abrupt onset of hypoxemia with diffuse pulmonary infiltrates, and the accumulation of a protein-rich pulmonary edema that causes a reduction in lung compliance, alveolar collapse, and ventilation-perfusion mismatch 5. Recent reports suggest that ion pump and channel functions are affected early in sepsis-induced ARDS 6 and that airway epithelial cells may be a valuable therapeutic target for ALI/ARDS treatment.

Pendrin is encoded by *SLC26A4* and acts as an anion exchanger that import Cl^-^ and export bases such as HCO_3_^-^, I^-^, OH^-^, SCN^-^, and formate 7-10. Pendrin is expressed in the inner ear and thyroid, and its mutation is associated with prelingual deafness (DFNB4) and Pendred syndrome 11,12. Normal airway epithelium shows negligible pendrin expression. However, the expression of pendrin is strongly up-regulated in inflammatory airway diseases, such as chronic obstructive pulmonary disease, allergic rhinitis, asthma 13,14; and enforced pendrin expression induces mucus overproduction with neutrophilic infiltration in mice airway epithelial cells 13. Madeo *et al.* reported that SLC26A4 mutation correlated with asthma resistance, although their results did not reach statistical significance due to the small number of patients 15. Pendrin upregulation is also observed in primary airway epithelial cells when they are cultured with IL-4, IL-13, and IL-17A 8,13,16. Nakagami *et al.* reported that pendrin-deficient mice had less allergen-induced airway hyper-reactivity and inflammation compared to control mice, and pendrin mRNA expression in human nasal epithelial cells increased in cases of common cold caused by rhinovirus 17. Interestingly, pendrin-null mice show reduced lung inflammation in response to *Bordetella pertussis* 18. This evidence indicates that pendrin is a critical protein in the pathogenesis of inflammatory airway disease. Recently, Dr. Verkman and his colleagues screened small molecules for pendrin inhibitors and showed that several pendrin inhibitor compounds significantly increased airway surface liquid thickness in cystic fibrosis patient bronchial epithelial cells expressing high levels of pendrin 19. We also previously performed a cell-based high throughput screening for the identification of small-molecule pendrin inhibitors; the pendrin inhibitor we screened (YS-01) showed a strongly therapeutic effect in an OVA-induced allergy asthma murine model, where it inhibited pendrin/OSCN^-^/NF-κB-mediated airway inflammation 20. Recently obtained data show that pendrin is also expressed in alveolar epithelia, and that administration of a non-specific anion exchanger inhibitor (methazolamide) attenuates the LPS-induced ALI phenotype 21.

Thus, we investigated whether the pendrin inhibitor YS-01 showed a therapeutic effect in an LPS-induced ALI mouse model and also examined the mechanism underlying the effects of YS-01. The findings of this study may provide a novel strategy for the treatment of inflammatory airway diseases including sepsis-induced acute lung injury.

## Materials and Methods

### Experimental animals

Wild-type male C57BL/6J mice, 8-10 weeks of age and weighing 20- 24 g, were purchased from Orient Bio (Sungnam, Republic of Korea). Pendrin knock-out (KO) mice used in the experiment were provided by JY Choi, and transgenic NF-κB reporter and SPC-Cre-ER^T2^ mice used for *in vivo* optical imaging (IVIS) were provided by KT Nam and BC Cho of Yonsei university (Table S1 and Figure S1).

### LPS-induced ALI in a murine model

LPS (Escherichia coli, O111: B4, Sigma) (10 mg/kg) in 50 μL PBS was administered by intranasal (i.n.) inhalation. The control group was given 50 μL of sterile PBS intranasally. For the pre-treatment model, YS-01 (10 mg/kg) in 50 μL DMSO was administered intra-peritoneally (i.p) 1 h before LPS inhalation. For the post-treatment model, two doses of YS-01 at 6 and 12 h after LPS inhalation were administered. The mice in the pretreatment group were euthanized and their lungs harvested 48 h after LPS inhalation. In the post-treatment group, euthanasia and sample collection occurred 24 h after the LPS administration. For the SCN^-^ experiment, 50 μL of NaOH, NaHCO_3_, or NaSCN (100 mM) were administered intranasally after YS-01 treatment. PBS was administered in the same manner for the control group. Additional details on the methods for animal experiments are provided in an online data supplement.

### Human bronchoalveolar lavage fluid collection

Forty-one patients with ARDS caused by pneumonia who underwent a bronchoalveolar lavage (BAL) were classified as the ARDS group. The 25 patients who were admitted for evaluation of solitary pulmonary nodule (SPN) without evidence of pulmonary infection were classified as the control group at the Severance Hospital between May 2013 and September 2015.

Prior to bronchoscopy, subjects were sedated with midazolam and fentanyl. The bronchoscope was inserted and wedged into the mouth for the BAL. BAL was performed following a standardized protocol (ARDS group: bronchus of pulmonary lesion, Control group: opposite bronchus from lung mass) and 10 cc of BALF was acquired from each patient using approximately 30 mL of 0.9% sodium chloride. Demographic and clinical data, including age, gender, body mass index (BMI), comorbidities, BALF analysis, cause of pneumonia, and final diagnosis were obtained from each participant, as well as medical records.

### Statistics

Statistical analysis was performed using Prism 5.0 (GraphPad Software). Group comparisons were performed using a two-tailed Student's t test to compare two groups, and a one-way ANOVA (followed by Bonferroni's multiple comparison post-hoc test) to compare more than two groups. Data are expressed as the mean ± SD. *P* values of less than 0.05 were considered statistically significant.

### Study approval

All animal protocols were approved by the Institutional Animal Care Committee of the Medical College of Yonsei University (2016-0322). Human study protocols were reviewed and approved by the Institutional Review Board of Yonsei University Health Service, Severance Hospital, Seoul, Korea (ARDS group IRB No. 4-2013-0585, control group IRB No. 4-2014-1014). Written informed consent was obtained from patients or their guardians regarding BALF sample use.

## Results

### LPS-induced ALI absent in pendrin-null mice

**Figure 1:**
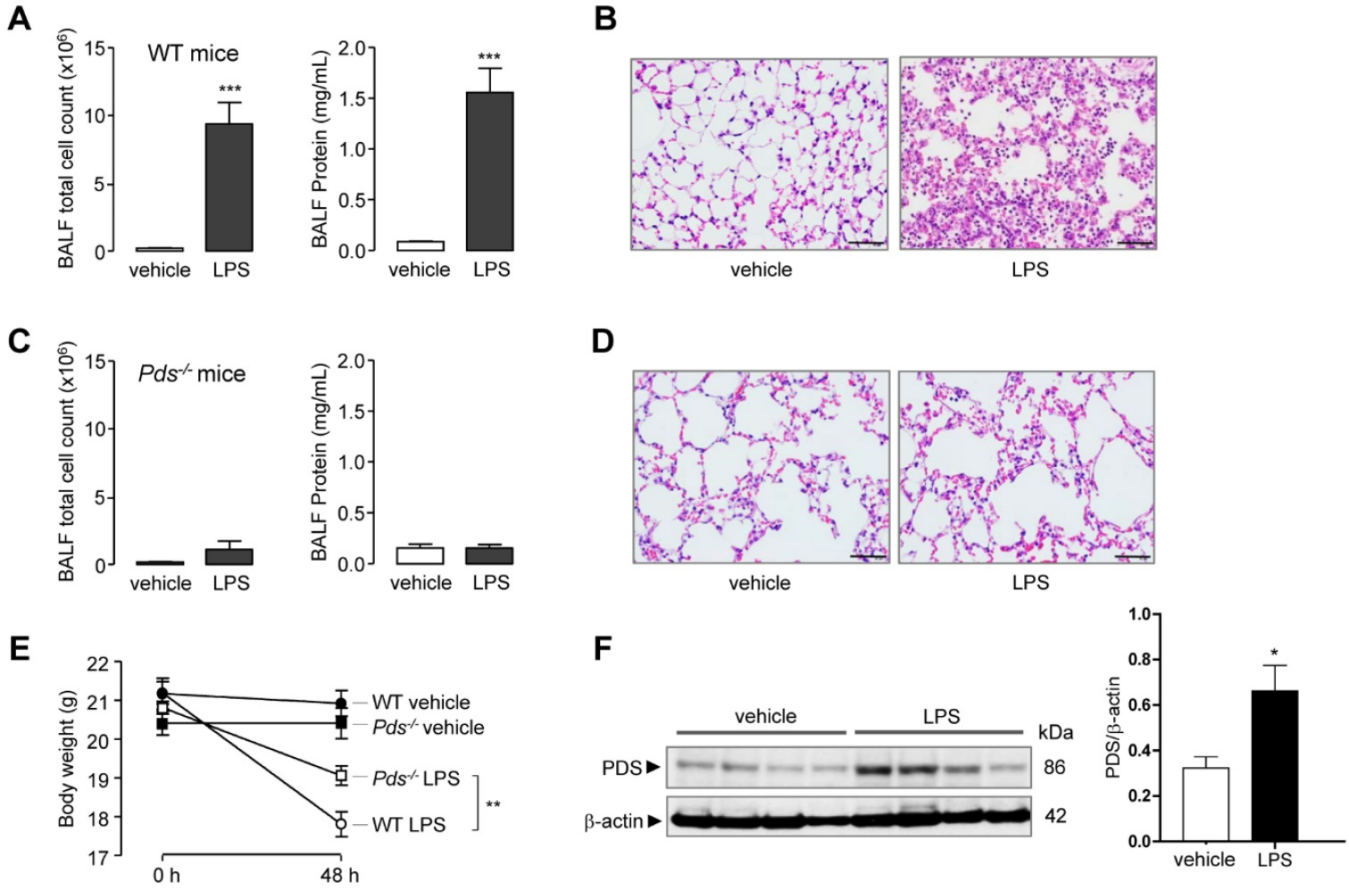
** Pendrin deficiency attenuated LPS-induced lung injury in mice.** Wild type (WT) and pendrin-null (*Pds^-/-^*) mice were intranasally administered LPS (10 mg/kg) or vehicle (PBS). (**A**) Total bronchoalveolar lavage (BAL) cell counts and BAL protein concentration were analyzed 48 h after LPS or PBS administration in WT mice. (**B**) Representative images of H & E staining of lung tissue 48 h after LPS or PBS administration (×400), scale bars: 50 µm. (**C**) Total BAL cell counts and BAL protein concentration were analyzed 48 h after LPS or PBS administration in pendrin-null mice. (**D**) Representative images of H & E staining of lung tissue 48 h after LPS or PBS administration (×400), scale bars: 50 µm. (**E**) Mice body weight changes. (**F**) Representative western blot analysis and densitometry of pendrin in lung lysates of LPS untreated and treated WT mice. Data provided are the mean ± SEM (n = 6-8 mice per group), *P < 0.05, **P < 0.01, ***P < 0.001 analyzed by Student's unpaired two-tailed t test.

As expected, intranasal LPS instillation induced ALI in WT mice. The total cell count and protein concentration in bronchoalveolar lavage fluid (BALF) was markedly increased after LPS treatment (Figure 1A). Lung histology also showed leukocyte infiltration and lung injury in WT mice (Figure 1B). In contrast, LPS did not increase cell count or protein concentration in pendrin-null mice (Figure 1C). Lung histology also revealed a lack of leukocyte infiltration and lung injury in pendrin-null mice after LPS treatment (Figure 1D). The mean body weight change after 48 hours of LPS instillation was more pronounced in WT mice than in pendrin-null mice (-3.38 g *vs.* -1.75 g, *P <* 0.01, Figure 1E). Immunoblot analysis of lung tissue lysates showed that pendrin protein expression was increased after LPS treatment compared to the vehicle control (Figure 1F). These results suggested that pendrin has an essential role in the development of LPS-induced ALI.

### The effect of a novel pendrin inhibitor (YS-01) in human alveolar epithelial cells

**Figure 2:**
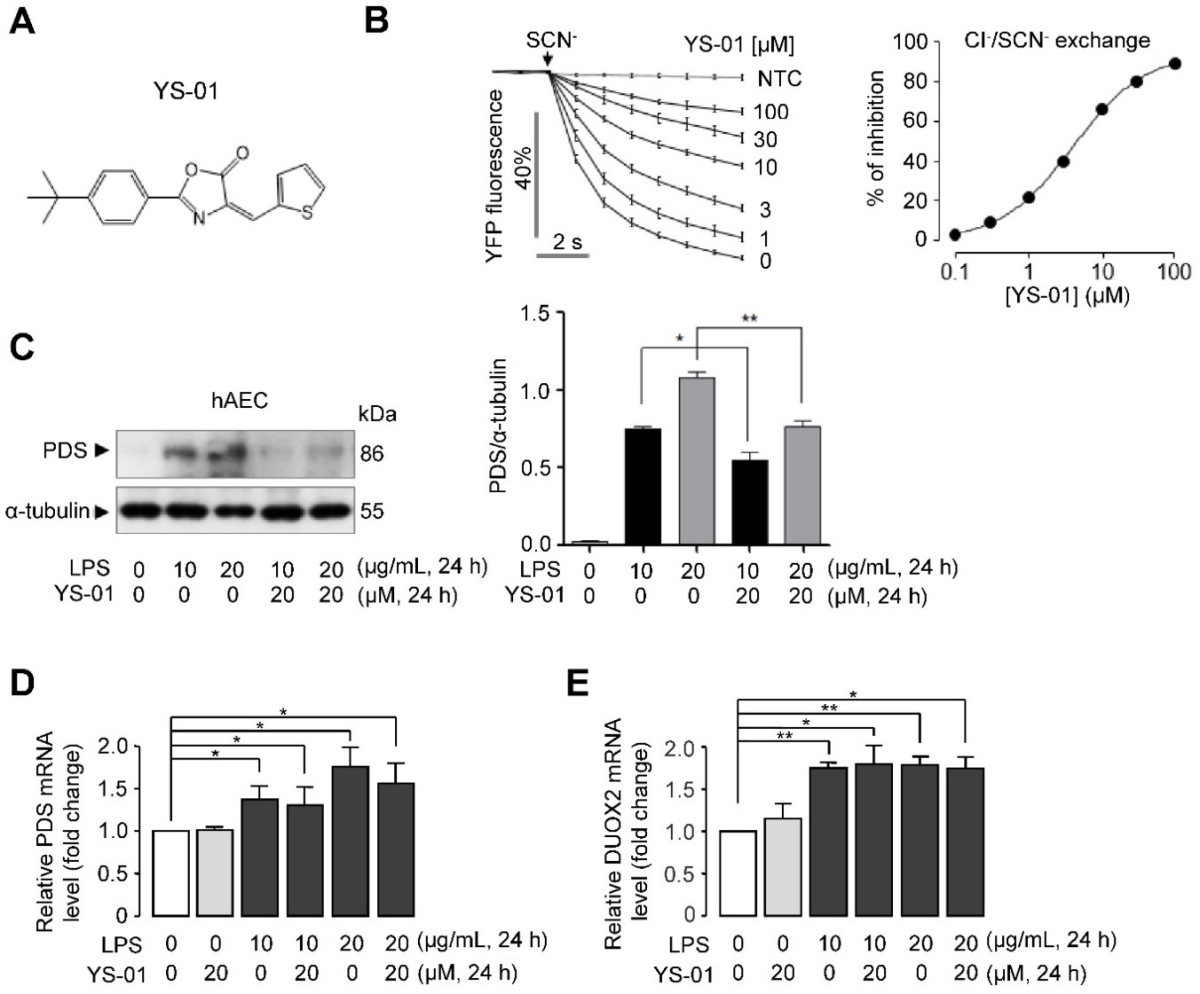
** Novel pendrin inhibitor (YS-01) blocked pendrin activity in human alveolar epithelial cells.** (**A**) Chemical structure of YS-01 pendrin inhibitor. (**B**) Inhibitory effect of YS-01 on human wild-type pendrin-mediated Cl^-^/SCN^-^ exchange activity in hAEC expressing human pendrin (mean ± SEM., n = 10). Indicated concentrations of YS-01 were pretreated for 10 min. Dose-response summary (right). (**C**) Representative western blot analysis of pendrin (PDS) in human alveolar epithelial cells (hAEC) and relative band intensity (mean ± SEM., n = 3). (**D**) PDS mRNA levels were determined by real-time quantitative PCR in hAEC (mean ± SEM., n = 3). (**E**) DUOX2 mRNA levels were determined by real-time quantitative PCR in hAEC (mean ± SEM., n = 3~4). *P < 0.05, **P < 0.01 analyzed by Student's unpaired two-tailed t-test.

In our previous study 20, a novel pendrin inhibitor, YS-01 was identified by the high-throughput screening of 54,400 synthetic compounds. YS-01 potently inhibited Cl^-^/I^-^, Cl^-^/SCN^-^, Cl^-^/HCO_3_^-^, and Cl^-^/OH^-^ exchange activity in naso-tracheal epithelia 20. In this study, we showed that YS-01 potently inhibited the Cl^-^/SCN^-^ exchange activity of pendrin (IC_50_ = 4.7 ± 0.82 μM) in pendrin-transfected human alveolar epithelial cells (hAEC) in a dose-dependent manner (Figure 2B). Our previous study showed that long-term treatment of YS-01 significantly reduced the protein expression level of pendrin without changing the mRNA expression level of pendrin 20. We also investigated the effect of LPS and YS-01 on protein and mRNA levels of pendrin in hAEC. Western blot analysis and densitometry showed that pendrin expression was increased after LPS treatment and suppressed after YS-01 treatment (Figure 2C). Meanwhile, LPS treatment significantly increased mRNA levels of pendrin, but YS-01 did not alter the mRNA levels of pendrin in hAEC (Figure 2D). Previous studies have shown that upregulation of pendrin can activate NF-κB by increasing hypothiocyanite (OSCN^-^) production via upregulation of dual oxidase (Duox1/Duox2) in airway epithelium of allergic inflammation 22. Real-time PCR analyses showed that LPS treatment significantly increased the mRNA levels of *Duox2*, and YS-01 did not alter mRNA levels of *Duox2* (Figure 2E).

### Pendrin inhibition attenuated LPS-induced ALI in mice

**Figure 3:**
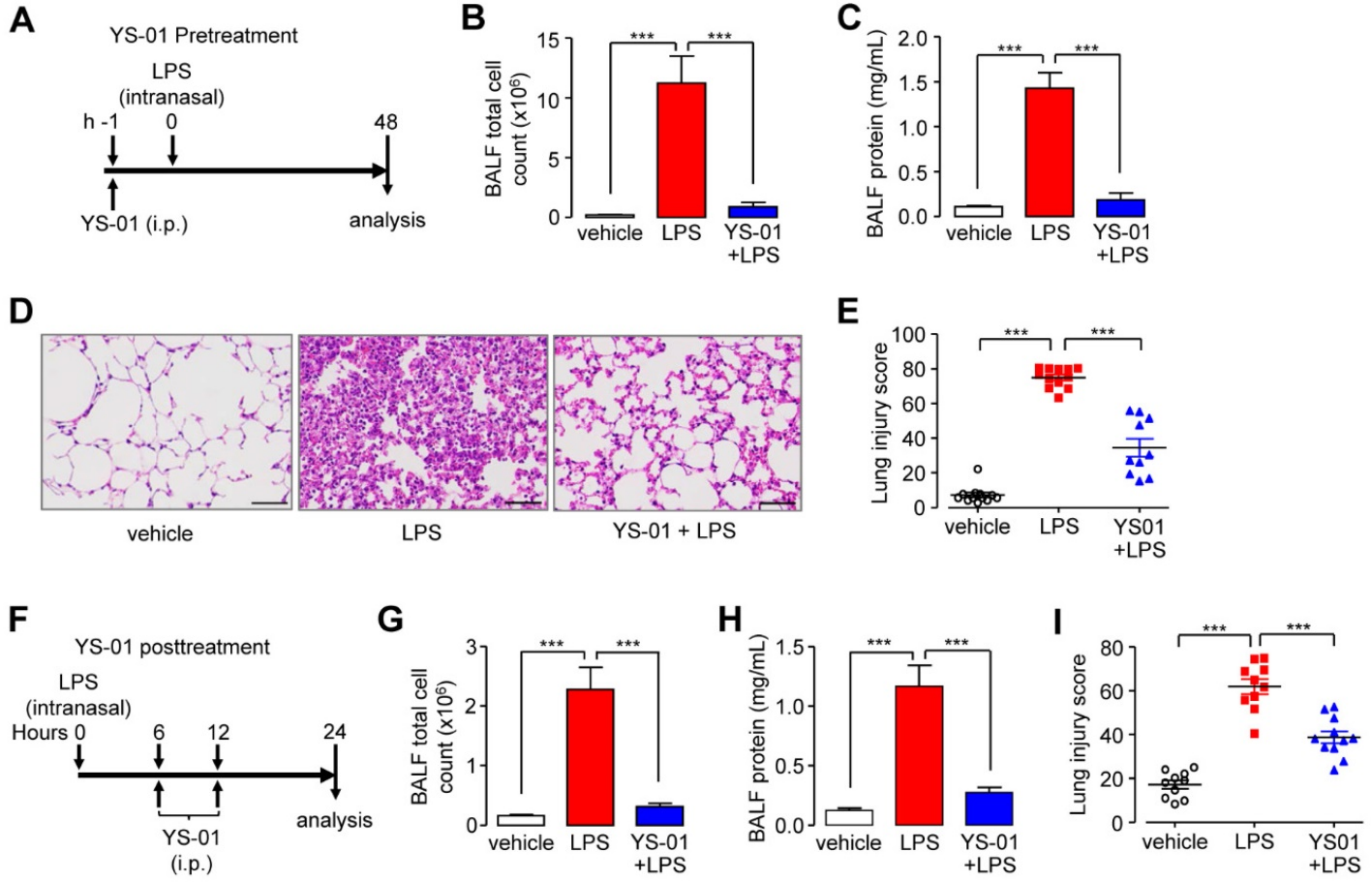
** YS-01 suppressed the LPS-induced acute lung injury phenotype in mice.** (**A**) YS-01 (10 mg/kg) was intraperitoneally injected 1 h before LPS treatment. (**B**) BALF total cell count. (**C**) BALF protein concentration. (**D**) Representative images of H&E lung tissue staining (×400), scale bars: 50 µm. (**E**) Lung injury scores. (**F**) YS-01 (10 mg/kg) was intraperitoneally injected at 6 and 12 h after LPS inhalation. (**G**) BALF total cell count. (**H**) BALF protein concentration. (**I**) Lung injury scores. Data provided are the mean ± SEM (n = 10-12 mice per group), ***P < 0.001, analyzed by one-way ANOVA with Bonferroni's post hoc test.

To investigate the protective function of YS-01 in an LPS-induced ALI mouse model, mice were treated with YS-01 one hour before LPS intranasal instillation and euthanized 48 hours after LPS administration (Figure 3A). YS-01 (10 mg/kg) pre-treated mice displayed decreased BALF total cell count and protein concentration levels compared to vehicle treated mice (Figures 3B and 3C). YS-01 pre-treatment also significantly reduced the lung injury score compared to vehicle-treated mice, where leukocyte infiltration occurred after LPS exposure (Figures 3D and 3E). To determine whether YS-01 treatment was effective after LPS injury, mice were treated with YS-01 at 6 and 12 h after LPS intranasal instillation, and then euthanized 24 hours after LPS administration (Figure 3F). YS-01 treatment after LPS instillation significantly reduced BALF total cell count and protein concentration, as well as lung injury score, consistent with the results of the YS-01 pre-treatment experiment (Figures 3G-I).

### SCN^-^ instillation triggered LPS-induced ALI in the presence of a pendrin inhibitor and in pendrin-null mice

**Figure 4:**
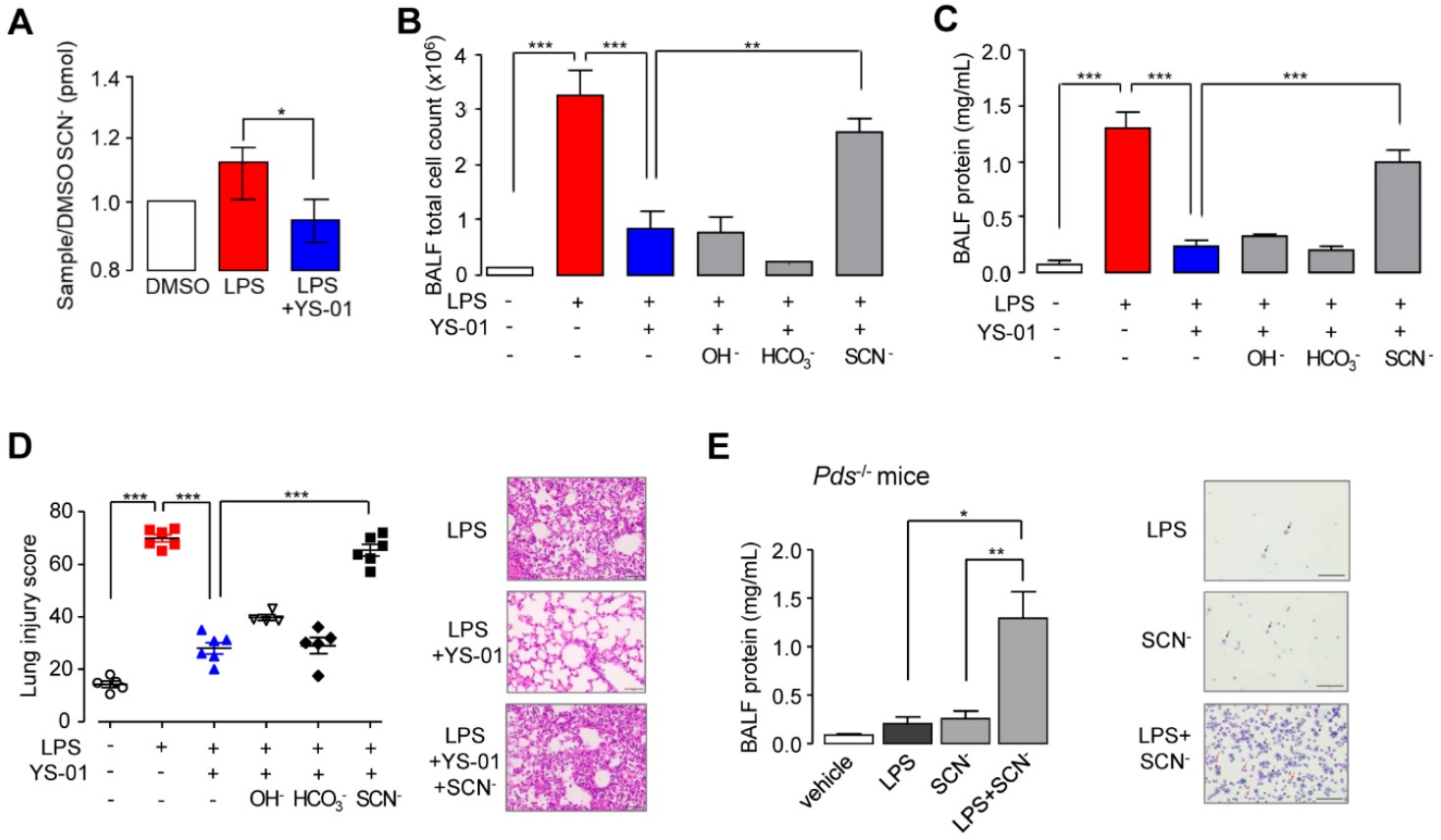
** SCN^-^ triggered LPS-induced lung injury in the presence of YS-01 or pendrin-null mice.** (**A**) Effect of YS-01 on SCN^-^ transport in HNE cells (mean ± SEM (n = 5 per group). (**B**) BALF total cell count. LPS (10 mg/kg, i.n.), YS-01 (10 mg/kg, i.p), NaOH (100 mM, i.n.), NaHCO_3_ (100 mM, i.n.) and NaSCN (100 mM, i.n.) were treated in WT mice. (**C**) BALF protein concentration. (**D**) Lung injury score. Representative lung tissues stained with H & E (right) (×400), scale bars: 50 µm. (**E**) BALF protein concentration in pendrin-null mice. NaSCN (100 mM, i.n.) was applied to the LPS treated pendrin-null mice. Representative mouse BALF cytospin stained with Diff-Quik Stain. Inflammatory cells, especially neutrophils (red arrows) were increased after LPS + NaSCN exposure compared to LPS or NaSCN alone (right). Black arrows represent macrophages (×200), scale bars: 100 µm. Data provided are the mean ± SEM (n = 5-6 per group), *P < 0.05, **P < 0.01, ***P < 0.001, analyzed by one-way ANOVA with Bonferroni's post hoc test.

To measure SCN^-^ transport, we used human nasal epithelial (HNE) cells using air-liquid interface culture. The SCN^-^ concentration at the apical surface was increased by LPS exposure and inhibited by YS-01 significantly (Figure 4A). To identify the underlying therapeutic effect mechanism of YS-01 in LPS-induced ALI, we supplied the anions secreted by pendrin (OH^-^, HCO_3_^-^, and SCN^-^). Intranasal application of NaSCN (50 μL of 100 mM) blocked the protective effects of YS-01 in LPS-induced ALI; BALF total cell count and lung injury score were increased compared to the group treated with LPS alone. However, administration of NaOH and NaHCO_3_ did not change the effect of YS-01 in LPS-induced ALI mice (Figures 4B and 4C). Histological analysis also revealed that the protective effect of YS-01 on inflammatory cell infiltration and lung injury after LPS administration was abolished by NaSCN administration (Figure 4D). More interestingly, simultaneous application of NaSCN with LPS induced robust lung injury in pendrin-null mice, whereas administration of LPS alone did not induce ALI (Figure 4E). These data strongly indicate that the therapeutic effect of YS-01 results from the SCN^-^ transport function inhibition of pendrin.

### Pendrin inhibitor blocked the NF-*k*B pathway and decreased inflammatory cytokines in an LPS-induced ALI mouse model

**Figure 5:**
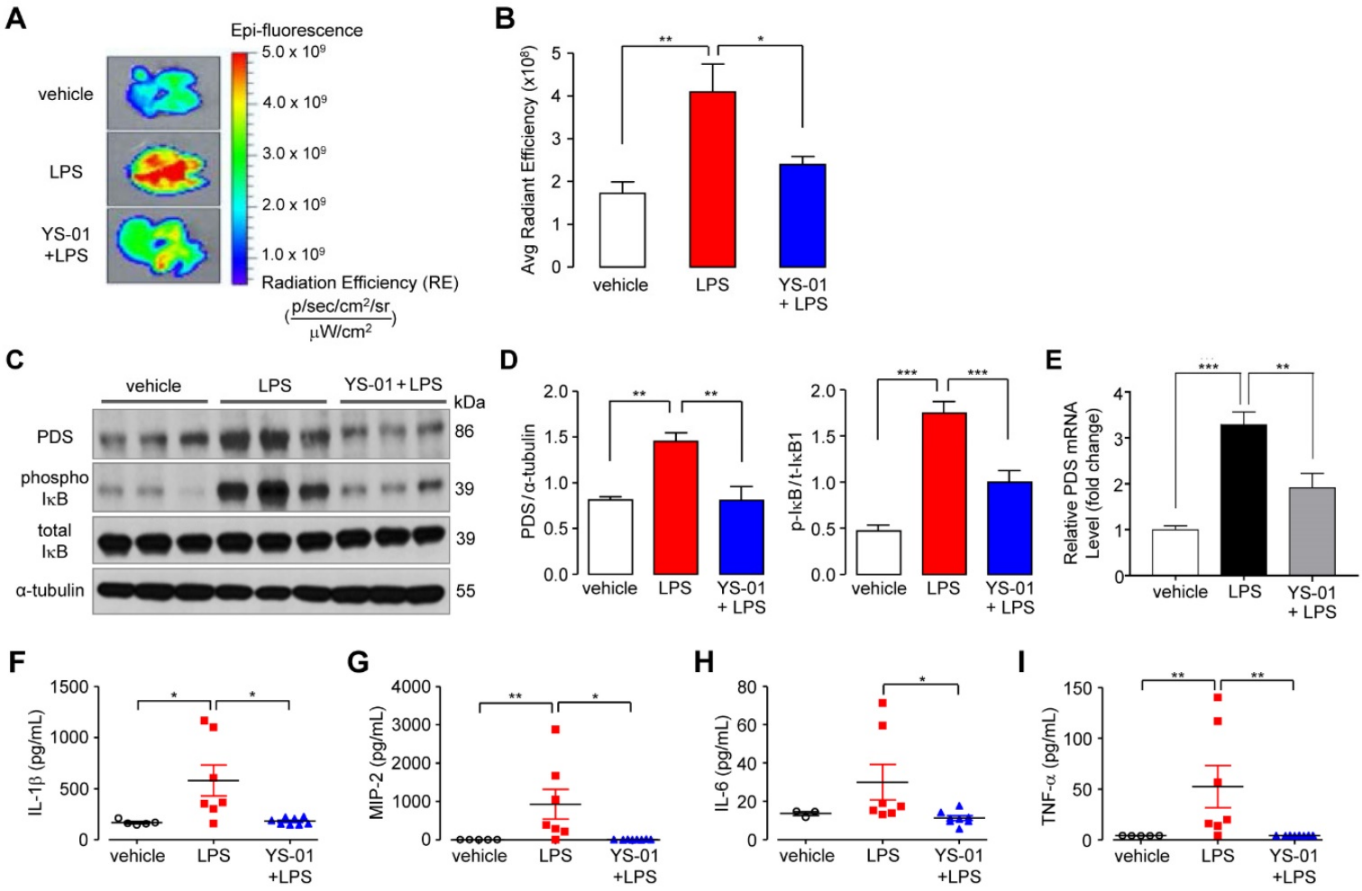
** YS-01 blocked the NF-*κ*B pathway and reduced the levels of proinflammatory cytokines in LPS-induced acute lung injury.** (**A**) Representative images of lungs from NF-κB/SPC-Cre mice exposed to LPS (10 mg/kg) and treated with either YS-01 (10 mg/kg) or a vehicle. IVIS image fluorescence is presented as the radiant efficiency. (**B**) Average fluorescence was quantified by region of interest analysis using Living Image software. Data provided are the mean ± SEM (n = 9-10 mice per group). (**C**) Representative western blot analysis in lung lysate. (**D**) Relative protein levels were measured by densitometry for pendrin and phospho-IκB. (means ± SEM, n = 6 per group). (**E**) Pendrin mRNA levels were determined by real-time quantitative PCR in lung tissue (means ± SEM, n = 11 per group). (**F-I**) IL-1β, CXCL2/MIP-2, IL-6 and TNF-α levels were measured by ELISA in lung tissue lysates. Data provided are the mean ± SEM (n = 7-8 mice per group), *P < 0.05, **P < 0.01, **P < 0.001, analyzed by one-way ANOVA with Bonferroni's post hoc test.

We further dissected the signaling pathway by which YS-01 acts in an LPS-induced ALI model using NF-*κ*B reporter/SPC-Cre-ER^T2^ mice. Quantitative fluorescence was determined using *in vivo* optical imaging (IVIS) images of mouse lungs that were aseptically removed immediately prior to imaging. Fluorescence in the excised lungs was increased after LPS treatment, which was suppressed by YS-01 (Figures 5A and 5B). These results suggested that NF-*κ*B activation in LPS instilled mice was suppressed by YS-01. Immunoblot analysis showed that the NF-*κ*B pathway was associated with YS-01 action. Phospho-I*κ*B protein expression, which represented NF-*κ*B activation, was increased after LPS administration, and YS-01 treatment before LPS significantly reduced phospho-I*κ*B expression (Figures 5C and 5D). Pendrin mRNA levels significantly increased after LPS and were decreased by YS-01 (Figure 5E). The levels of cytokines including IL-1ß, tumor necrosis factor-α, and macrophage inflammatory protein (MIP)-2 were significantly increased after LPS administration compared to PBS (Figures 5F-I). IL-6 tended to be increased, relative to PBS, although the difference was statistically insignificant. In contrast, levels of pro-inflammatory cytokines decreased in YS-01 pre-treated mice compared to those that received vehicle (DMSO) treatment after LPS administration (Figures 5F-I).

### Pendrin levels in human BALF were increased in patients with pulmonary infection

**Figure 6:**
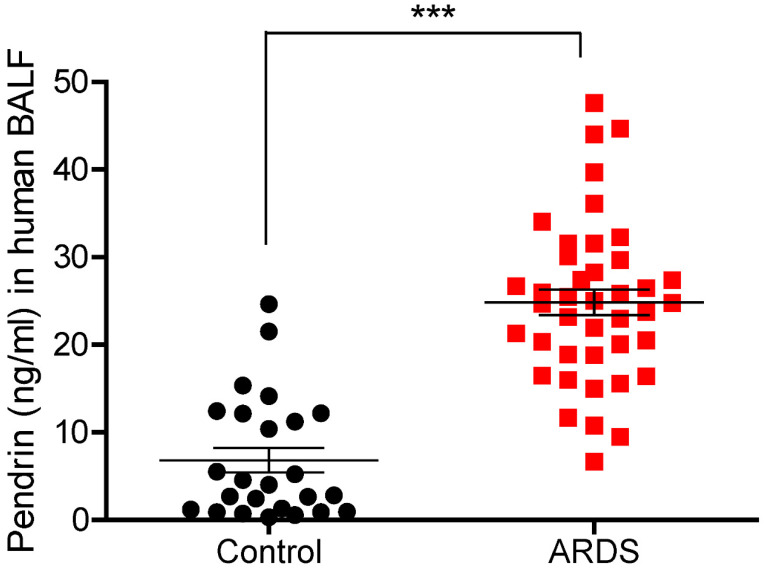
** Pendrin levels of human BALF.** Patients with ARDS caused by pneumonia displayed increased pendrin levels compared to the control patients (without infection). Pendrin levels were measured from human BALF supernatant by ELISA (Control n = 25, ARDS n = 41). *P < 0.05, **P < 0.01, ***P < 0.001, analyzed by Student's unpaired two-tailed t test.

To translate *in vitro* and *in vivo* findings to human disease, we measured BALF pendrin protein expression in patients with ARDS caused by pneumonia (ARDS group, n = 41) and patients with a solitary pulmonary nodule (SPN) but no infection (control group, n = 25). Patient clinical characteristics are shown in Table 1. Mean age was not significantly different between the control and ARDS groups (63.8 vs. 65.9, *p* = 0.517) and males were predominant in both groups (80% vs. 78%, *p* = 0.851). Among the ARDS patients, the median length of hospital stay was 36 days, and the 28-day mortality rate was 24.4% (Table 1). Detailed clinical information of study patients can be found online in Table S2. Pendrin level was significantly elevated in the BALF of ARDS patients (n = 41) compared to that of the control subjects (n = 25) (mean, 24.86 vs. 6.83 ng/mL, *P <* 0.001) (Figure 6).

## Discussion

Emerging evidence strongly suggests that pendrin is a key protein in the development of airway inflammatory diseases including asthma, chronic obstructive pulmonary disease, and rhinitis 13,23. We demonstrated that the pendrin expression level in LPS-treated mouse airways increased. Moreover, we showed that LPS-induced ALI did not develop in pendrin null mice, which strongly indicated the critical role of pendrin in ALI pathogenesis. This is consistent with a recent report that showed pendrin expression was enhanced in LPS-induced ALI, and a non-specific pendrin inhibitor attenuated ALI in mice 21. This evidence encouraged us to develop a pendrin inhibitor as a novel drug for ALI treatment. We screened more than 54,400 small molecules and found a specific pendrin inhibitor (YS-01) that did not affect other ion transports, such as Cystic fibrosis transmembrane conductance regulator (CFTR) and calcium-activated chloride channel (CaCC) 20. Pendrin expression was upregulated by LPS treatment in human alveolar epithelial cell culture, which was effectively suppressed by YS-01. Figures 2C-2D show that treatment of YS-01 significantly reduced the protein expression of pendrin without changing mRNA expression in hAEC. These results suggested that YS-01 decreased the protein stability of pendrin and consistent with our previous results in human nasal epithelial cells 20. Interestingly, YS-01 reduced both protein and mRNA levels of pendrin in the animal model of LPS-induced lung injury (Figures 5D and 5E). The reduction of pendrin mRNA expression levels in YS-01-treated mice may be due to the decreased inflammatory signals by inhibition of pendrin*.* Decreased pendrin mRNA expression and destabilization of pendrin protein by YS-01 may enhance the reduction of pendrin protein *in vivo.* Surprisingly, YS-01 almost completely prevented the development of LPS-induced ALI in mice. Furthermore, administration of YS-01 after LPS treatment attenuated lung injury in mice, which indicated that the clinical therapeutic window of pendrin inhibitors is wide enough to include post-ALI periods. We demonstrated that pendrin expression in BALF from pneumonia patients and LPS-treated mouse airways increased, which strongly suggested a high possibility for the clinical application of pendrin inhibitors in inflammatory airway disease.

**Figure 7:**
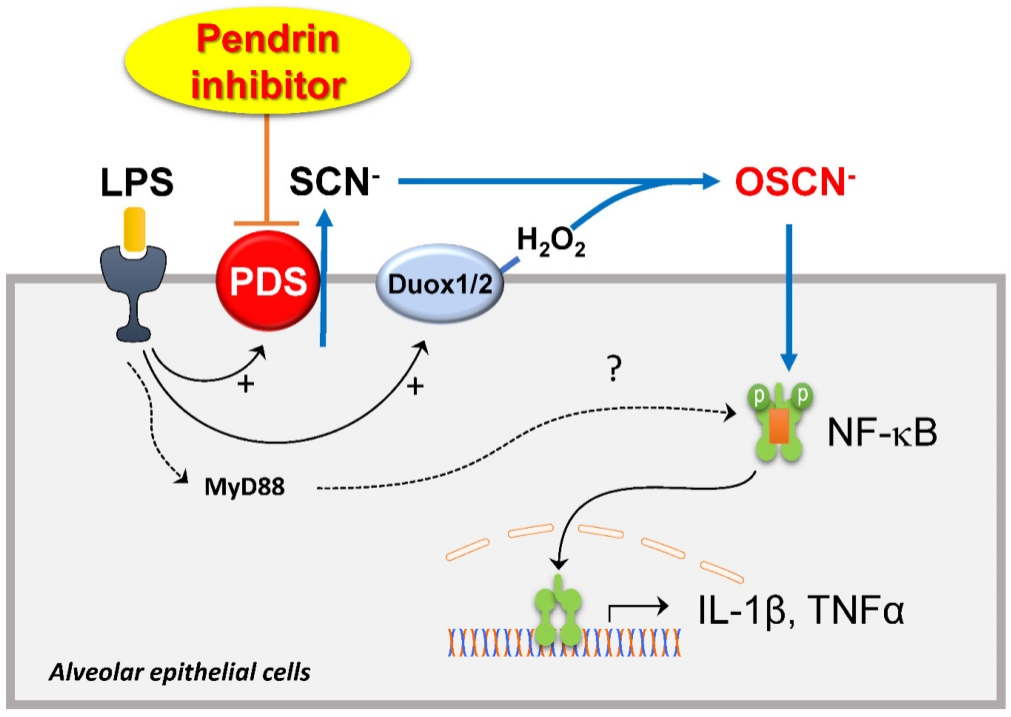
** Schematic diagram of the roles of pendrin and its inhibitor in LPS-induced lung injury.** SCN^-^ is actively transported into pulmonary lumens via pendrin at the apical surface of the alveolar epithelia. SCN^-^, together with H_2_O_2_, is catalyzed into OSCN^-^ by peroxidases. The produced OSCNˉ activates NF*-κ*B and causes inflammatory cytokine release, neutrophil infiltration, and subsequent lung injury. Pendrin inhibitor YS-01 blocks the transepithelial transport of SCN^-^ that inhibits the OSCN^-^ induced NF*-κ*B activation and subsequent onset of ALI.

The role of pendrin and the mechanism underlying the therapeutic effects of YS-01 in the ALI model are unclear. We focused on the Cl^-^/SCN^-^ exchange activity of pendrin and hypothiocyanite (OSCN^-^), which is synthesized from SCN^-^ transported via several anion transporters (including pendrin) by lactoperoxidases in the airway epithelia. OSCN^-^ is known to be part of an important innate defense system against microbes in the airways 24,25 and also induces airway inflammation in airway epithelia. Recent studies show that IL-4 upregulates the Cl^-^/SCN^-^ exchange activity of pendrin and increases OSCN^-^ production, which results in NF-κB activation and induces airway inflammation in a murine allergic asthma model 22,26. We showed that the therapeutic effect of YS-01 on lung injury disappeared when NaSCN was added into the airways of mice. NaSCN application also induced lung injury even in pendrin null mice, whereas LPS-induced ALI had not developed. These data indicate that airway surface SCN^-^ transported by pendrin is an essential component for LPS- induced airway inflammation. NF-κB is a crucial transcription factor for inflammatory responses in airways 27,28 and its inhibition attenuates ALI *in vivo* 29,30. We also observed that YS-01 inhibited LPS-induced NF-κB activation and subsequent cytokine production in a murine ALI model and alveolar epithelia. Collectively, our data indicated that the pendrin inhibitor mode of action resulted from YS-01 blocking the transepithelial transport of SCN^-^, and subsequently inhibiting OSCN^-^ generation and NF-κB activation. This resulted in the suppression of proinflammatory cytokine production (Figure 7). This is a very similar mode of action to that uncovered in our asthmatic mouse model, where pendrin inhibitors attenuated OVA-induced allergic airway inflammation by inhibiting the pendrin/OSCN^-^/NF-κB cascade 20. However, if we take into account the previous reports 31,32, which showed that LPS can also activate NF-κB via the TLR4/MyD88 pathway, the reason for YS-01 almost completely suppressing LPS-induced ALI remains unknown. However, the deficiency of ALI phenotypes triggered by NaSCN in pendrin null mice strongly indicates that pendrin-mediated OSCN^-^ dominantly activates the NF-κB cascade in an LPS-induced ALI model.

Although critical care for ALI patients has improved, ALI/ARDS mortality remains high and there are limited options for the medical treatment of ALI/ARDS. Because YS-01 showed a strong therapeutic effect on our ALI murine model, pendrin could be a novel target for the medical treatment of ALI/ARDS. It is promising that pendrin expression was upregulated in pneumonia patient BALF, which increases the potential clinical therapeutic benefit of a pendrin inhibitor for ALI/ARDS. However, there are still several issues requiring resolution before the clinical application of pendrin as a drug for ALI/ARDS treatment. First of all, the systemic adverse effect of pendrin inhibitors must be ruled out in areas where pendrin is expressed, including the inner ear, thyroid, and kidney. Although we can rule out hearing loss and hypothyroidism in a mouse model based on our preliminary studies 20, we must examine possible systemic side effects more thoroughly. We can also avoid potential systemic side effects by the local administration of pendrin inhibitors in patients with ALI. Another important issue for the development of pendrin inhibitors as a drug for ALI treatment is the specificity of YS-01 on pendrin. Our previous study 20 showed that YS-01 weakly stimulates SLC26A3 (DRA) and SLC26A6, so it can exert a biological effect in the intestine and kidney. We need to find more specific YS-01 analogues through additional structure-activity relationship analysis. Nevertheless, YS-01 is chemically stable with low cytotoxicity and works at a nanomolar level; it is an excellent compound for the further development of a final candidate for clinical trials.

Although our current study suggests that the inhibition of SCN^-^ transport by pendrin inhibitor is a crucial mechanism, we cannot demonstrate the difference in SCN^-^ concentration in mice airway space. Further study is needed to figure out a proper method to quantify SCN^-^ or OSCN^-^ levels directly. Despite such limitations, we demonstrated that pendrin is essential for LPS-induced ALI, and a small molecule (YS-01) that inhibits pendrin strongly suppressed LPS-induced ALI. Our study indicates that pendrin inhibitors are a promising new drug class for ALI treatment.

## Supplementary Material

Supplementary figures and tables.

## Figures and Tables

**Table 1:** Clinical characteristics of study patients

	Control (n = 25)	ARDS* (n = 41)	*P*
Age, years, mean ± SD	63.8 ± 9.7	65.9 ± 13.6	0.517
Gender, male, N (%)	20 (80.0)	32 (78.0)	0.851
BMI (kg/m^2^), mean ± SD	24.5 ± 4.3	22.6 ± 3.0	0.040
ICU admission, N (%)	0	41 (100)	-
Intubation/ARDS, N (%)	0	41 (100)	-
P/F ratio, mean ± SD	-	157.3 ± 52.8	-
Bacteremia, N (%)	0	9 (22.0)	-
Length of stay, d, median (IQR)	2 (1-2)	36 (26-57)	-
28-day mortality, N (%)	0	10 (24.4)	-
In-hospital mortality, N (%)	0	28 (68.3)	-
Pendrin level, ng/mL, mean ± SD	6.83 ± 6.91	24.86 ± 9.28	<0.001

Values are presented as the mean±SD, median (interquartile range, IQR), or number (%); *ARDS due to pneumonia; Abbreviations: BMI, body mass index; ICU, intensive care unit; ARDS, acute respiratory distress syndrome; P/F, PaO_2_/FIO_2_.

## Abbreviations

ALI: acute lung injury
ARDS: acute respiratory distress syndrome
BALF: bronchoalveolar lavage fluid
BMI: body mass index
CaCC: calcium-activated chloride channel
CFTR: cystic fibrosis transmembrane conductance regulator
hAEC: human alveolar epithelial cells
ICU: intensive care unit
IVIS: *in vivo* optical imaging
LPS: Lipopolysaccharide
SPN: solitary pulmonary nodule
WT: wild type
