# Supplementary material for: Inhibition of Pendrin by a small molecule reduces Lipopolysaccharide-induced acute Lung Injury
Source: Theranostics. 2020 Aug 7;10(22):9913–22. doi: 10.7150/thno.46417 (PMC7481407; doi:10.7150/thno.46417)
Supplement: Supplementary file 1 — Supplementary figures and tables. [file thnov10p9913s1.pdf]

Online Data Supplement

Inhibition of pendrin by a small molecule reduces lipopolysaccharide-induced acute lung injury

Eun Hye Lee[1], Mi Hwa Shin[1], Mia Gi[2,3], Jinhong Park[4,5], Doona Song[6], Young-Min Hyun[7], Ji-Hwan Ryu[8,9], Je Kyung Seong[10,11], Yoon Jeon[12], Gyoonhee Han[4,6], Wan Namkung[4,5*], Moo Suk Park[1*], and Jae Young Choi[2,3*]

Supplemental Materials

Materials & Methods

All animal experiments were conducted in accordance with the recommendations in the Guide for the Care and Use of Laboratory Animals of the National Institutes of Health.

Transgenic Experimental animals

Pendrin knock-out (KO) mice used in the experiment were provided by JY Choi of Yonsei University (Supplementary Table S1). Transgenic NF-κB reporter/SPC-Cre-ER^{T2} mice were used for IVIS in this study. Briefly, NF-κB reporter mice contain a ROSA26 lox-STOP-lox-cassette inserted between a promoter and the NF-κB-luciferase-dTomato gene (Supplementary Figure S1). Normally, the stop gene is located between loxP and loxP, and NF-κB-luciferase-dTomato is not expressed. The surfactant protein C (SPC)-Cre-ER^{T2} mice were bred with ROSA26R mice to obtain NF-κB reporter/SPC-Cre-ER^{T2} mice. Cre-ER^{T2} recombinase activity in these transgenic mice was induced by tamoxifen [1,2]. These NF-κB reporter/SPC-Cre-ER^{T2} mice express NF-κB activity in the alveolar epithelium either through dTomato fluorescence or luciferase in the presence of tamoxifen. Increased fluorescence in IVIS image means increased NF-κB activity in the alveolar epithelium. Tamoxifen (Sigma, USA) was dissolved in a 10:1 sunflower seed oil/ethanol mixture (10 mg/mL). Each 4-week-old mouse was injected intraperitoneally with 100 mL of tamoxifen/day for 5 consecutive days. One week after the last injection, mice were used for IVIS or intravital imaging. Transgenic NF-κB reporter and SPC-Cre-ER^{T2} mice were provided by KT Nam and BC Cho of Yonsei University (Table S1).

Isolation of bronchoalveolar lavage

All mice were euthanized by a lethal overdose of ketamine and xylazine. BALF was obtained by tracheal cannula using 1 mL sterile saline. The BALF was centrifuged (4°C, 3000 rpm, 10 min) and the supernatant was stored at −80°C for further analysis. The cell pellet was reconstituted in 100 μL PBS and used for cell counts and cytospin samples. Total cell numbers in each sample were determined using a hemocytometer (Marienfield) according to the manufacturer's protocol. A 90 μL aliquot of each sample was transferred into the slide chambers, which were then inserted into a cytospin with the slide facing outward. Slides were centrifuged at 800 rpm for 5 min, then removed from the cytocentrifuge and dried prior to staining. Cytospins were prepared with a cytocentrifuge (Shandon Cytospin 4 cytocentrifuge, Thermo Scientific, Waltham, MA, USA) and stained with Diff-Quik Stain Set (Dade Behring, Newark, DE, USA) to assess inflammation. The protein concentrations of the BAL supernatant were determined using a BCA assay (Thermo Fischer Scientific). Two microliters of each sample and 198 μL of working reagent were pipetted into a microplate well and mixed thoroughly on a plate shaker for 30 s. After incubation for 30 min at 37°C, the plate was cooled and the absorbance read at 562 nm in a spectrophotometer.

Lung tissue harvest and histological examination

The right lung was isolated and stored at −80°C prior to protein extraction after flushing the pulmonary vasculature with saline under low pressure. The left lung was inflated via a tracheotomy with low-melting point agarose (4%) in PBS at 25 cm H_2O pressure until the pleural margins sharpened. The lungs were then excised and fixed overnight in 10% formaldehyde in PBS and embedded in paraffin for 5 μm sectioning. Left lung sections were stained with H & E and subjectively evaluated by light microscopy. Histopathology was reviewed in a blinded manner by two qualified investigators (EH Lee and MS Park). Five easily identifiable pathological processes were scored using the weighted scale presented in the official ATS workshop report [3]. Lung sections

were processed for immunohistochemistry using anti-rabbit SLC26A4 (ab98091, abcam) antibody.

Measurement of Cl^-/SCN^- exchange

The human alveolar epithelial cells (hAEC) purchased from Science Cell (Catalog #3200), which consisted of alveolar type I and type II epithelial cells, lined more than 99% of the internal surface area of the lung. The hAEC from Science Cell research Laboratories were isolated from human lung tissues, cryopreserved at P0, and delivered frozen. The hAEC transient transfected human pendrin (PDS) and YFP-F46L/H148Q/I152L were plated in 96-well plate at a density of 2×10^4 cells per well and incubated for 48 h. Each well of the 96-well plate was washed two times with 200 μL of PBS, and it was filled with 100 μL of PBS. To measure the effect of YS-01 on hPDS-mediated Cl^-/SCN^- exchange activity, cells were pre-treated with YS-01. After 10 min of incubation at 37°C, the 96-well plate was placed on the stage of an inverted fluorescence microscope (Nikon, Tokyo, Japan) equipped with a cooled charge-coupled device camera (Zyla sCMOS), image acquisition and analysis software (Meta Imaging Series 7.7). Each well was assayed individually for hPDS-mediated SCN^- influx by recording YFP fluorescence continuously (2 s per point) for 4 s (baseline). Then, 100 μL of 140 mM SCN^- solution was added at 4 s and then YFP fluorescence was recorded for 14 s. To investigate the effect of long-term treatment of YS-01 in LPS exposed hAEC, we treated the hAEC with LPS (10 μg/ml or 20 μg/ml) for 24 hours with or without YS-01 (20 μM/ml).

Transepithelial SCN^- transport

Human nasal epithelial (HNE) cells were plated on transwell permeable supports and cultured at the air-liquid interface for 14 days. Complete differentiated HNE cells were incubated with LPS (10 μg/ml) or DMSO (control) for 24 hours with or without YS-01 (20 μM/ml). The basolateral side was treated with 1 mL of PBS containing 10 mM glucose and 5 μCi of $S^{14}CN$ (total concentration of SCN^-:

86 μM). The apical side of fluid was collected and placed in scintillation vials for the evaluation of radioactivity.

Real-time RT-PCR analysis

Total messenger RNA (mRNA) was extracted using TRIzol reagent (Invitrogen, Carlsbad, CA, USA) and reverse-transcribed using random hexamer primers, an oligo (dT) primer, and SuperScript® III Reverse Transcriptase (Invitrogen). Quantitative real-time PCRs were performed using the StepOnePlus Real-Time PCR System (Applied Biosystems, Foster City, CA, USA) and Thunderbird SYBR qPCR mix (Toyobo, Osaka, Japan). The thermal cycling conditions included an initial step of 95°C for 5 min followed by 40 cycles of 95°C for 10 s, 55°C for 20 s, and 72°C for 10 s in a 96-well reaction plate. The primer sequences are described in Table E3.

ELISA

Macrophage inflammatory protein (MIP-2), interleukin-1β (IL-1ß), interleukin 6 (IL-6), and tumor necrosis factor α (TNF-α) levels in lung lysates were measured using ELISA kits (Millipore) according to the manufacturer's directions. Human BALF was centrifuged (10 min; 1500 *g*) and the supernatant was cryopreserved at −80°C until use. Pendrin levels of human BALF were measured using human SLC26A4 ELISA kits (MBS764789, Mybiosource) according to the manufacturer's directions.

Western blotting

Lung tissues were harvested and lysed in homogenization buffer (PRO-PREP^{TM} Extraction solution, iNtRON Biotechnology). The samples were centrifuged at 13000 *g* for 30 min at 4°C. Supernatant

protein concentration was determined by BCA assay (Thermo Fisher Scientific). Equal amounts of protein were separated by SDS/PAGE and transferred to a nitrocellulose membrane. Membranes were blocked with 5% skim milk in TBS-T (TBS (170-6435, Bio-Rad Laboratories) and 1% Tween-20 (170-6531, Bio-Rad Laboratories) for 1 h at room temperature. Membranes were then incubated overnight with primary antibody diluted in 5% skim milk in TBS-T at 4°C. After washing with TBS-T, the blots were incubated with horseradish peroxidase-conjugated secondary antibodies and 5% skim milk in TBS-T for 1 h at room temperature, then developed using a Super-Signal West Pico chemiluminescence detection kit (Pierce). The antibodies used in the present study included rabbit SLC26A4 (ab98091, abcam), mouse phospho-IκB (9246, Cell Signaling Technology), mouse IκB (4814, Cell Signaling Technology), and rabbit α-tubulin (PA5-16891, Cell Signaling Technology). Western blot quantification was conducted using ImageJ (Image Processing and Analysis in Java; NIH, USA) software.

IVIS (in vivo optical imaging)

Imaging of live animals and organs was performed using an IVIS kinetic imaging system (Caliper Life Sciences, Preston Brook Runcorn, UK). The IVIS system consisted of a cooled charge-coupled device camera mounted onto a light-tight specimen chamber. The fluorescent excitation light was provided by a halogen lamp in combination with appropriate excitation filters. Emission filters were placed in front of the camera aperture to allow recording of specific wavelengths of light, depending on the emission spectra of the FP examined. Fluorescence imaging was obtained with an excitation wavelength of 554 nm and emission wavelength of 581 nm (dTomato). Mice were divided into three groups for IVIS imaging, (DMSO + PBS, DMSO + LPS, and YS-01 (10 mg/kg, i.p) + LPS), and *ex vivo* lungs were aseptically removed 6 h after LPS treatment. When organs were imaged, they were placed as flat as possible to allow full and consistent light penetration in order to minimize potential variation in the measurements due to differing tissue thicknesses. Fluorescence was quantified using

the region of interest tool in Living Image software (version 3.2, Caliper Life Sciences).

SUPPLEMENTARY FIGURE LEGENDS

Figure S1. NF-*κ*B reporter mouse

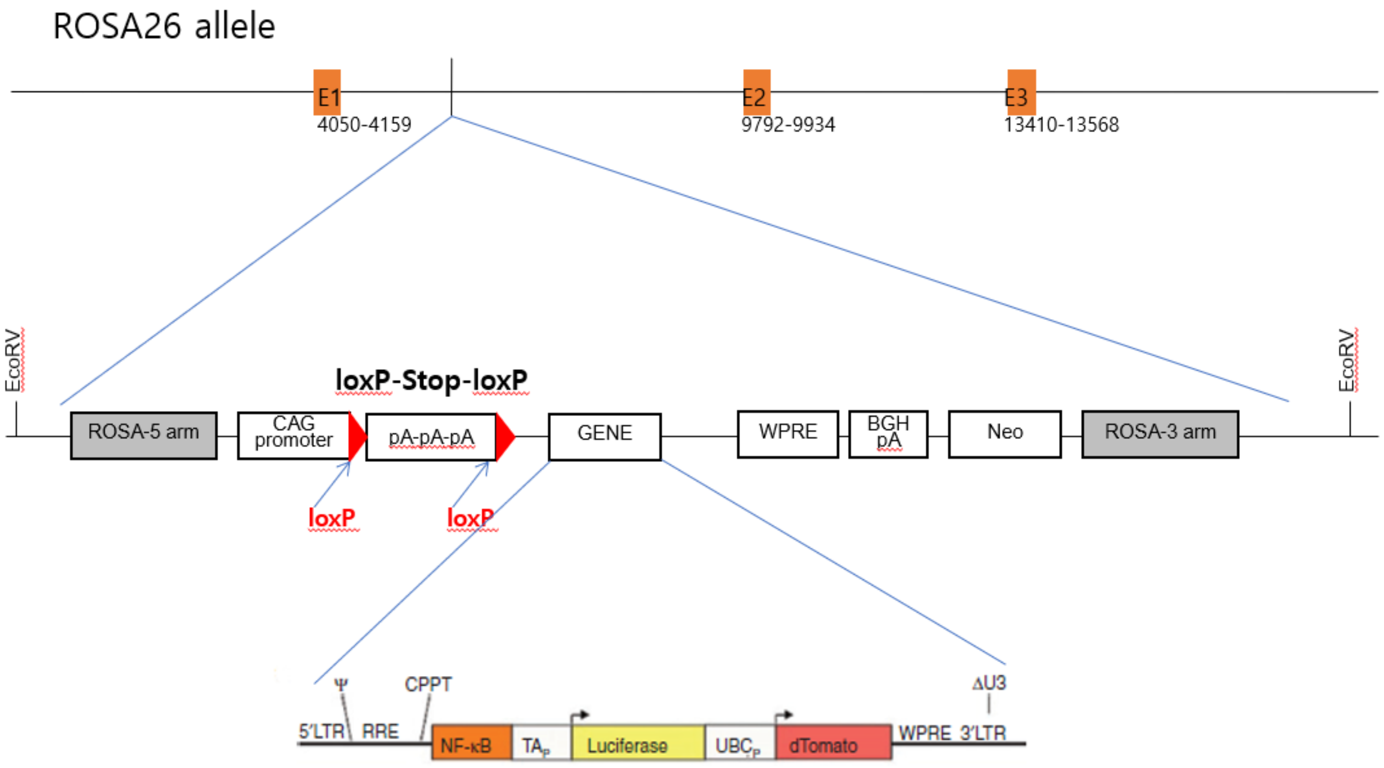

SUPPLEMENTARY TABLE LEGENDS

Table S1. Mouse genotyping PCR Primers

**Mouse genotyping primers**		**Sequence**
**Pendrin KO mouse**		
pPNTloxP (22)		GGG TGC GGA GAA AGA GGT AAT G
exon8 (25)		GCA TTG TAG TTC TTT TCC AAG TTG G
exon7 (18)		TGC CGA TTT CAT CGC TGG
**NF-κB reporter mouse**		
ROSA	ROSA 1 (forward-common)	AAA GTC GCT CTG AGT TGT TAT
	ROSA-2 (reverse-WT)	GGA GCG GGA GAA ATG GAT ATG
	ROSA (reverse-mut)	GGC GGG CCA TTT ACC GTA AG
Insert	NFκB_For	GAT CCC CAT CAA GCT GAT CCG G
	NFκB_Rev	GCT GCG AAA TGC CCA TAC TG
SPC-CreER^{T2}		
Forward		GTC GAT GCA ACG AGT GAT GA
Reverse		TCA GCT ACA CCA GAG AC

Table S2: Baseline characteristics of all human subjects enrolled in the current study

	Final diagnosis	Age	Sex	BMI	Pneumonia/ Sepsis	ARDS	P/F ratio	Cause of Pneumonia	Time from intubation to BAL (days)	Hospital stay (days)	Mortalities		BALF analysis			
											In-hospital	28-day	WBC	PMN (%)	Lympho (%)	Mono (%)
1	Benign nodule	67	M	23.3	0	0	—	—	—	3	0	0	—	—	—	—
2	Ewing sarcoma	52	M	21.1	0	0	—	—	—	2	0	0	—	—	—	—
3	Benign nodule	69	M	25.1	0	0	—	—	—	3	0	0	—	—	—	—
4	Lung cancer	74	M	18.0	0	0	—	—	—	3	0	0	—	—	—	—
5	Benign nodule	79	M	26.8	0	0	—	—	—	2	0	0	—	—	—	—
6	Lung cancer	73	M	35.4	0	0	—	—	—	2	0	0	—	—	—	—
7	Benign nodule	44	F	19.1	0	0	—	—	—	2	0	0	—	—	—	—
8	Benign nodule	67	M	23.1	0	0	—	—	—	2	0	0	—	—	—	—
9	Benign nodule	61	M	21.8	0	0	—	—	—	2	0	0	—	—	—	—
10	Lung cancer	62	M	20.4	0	0	—	—	—	2	0	0	—	—	—	—
11	Mild bronchitis	64	M	26.1	0	0	—	—	—	1	0	0	—	—	—	—
12	Benign nodule	73	M	24.9	0	0	—	—	—	2	0	0	—	—	—	—
13	Benign nodule	81	M	24.2	0	0	—	—	—	2	0	0	—	—	—	—
14	Lung cancer	51	F	19.5	0	0	—	—	—	2	0	0	—	—	—	—
15	Focal inflammation	58	F	22.4	0	0	—	—	—	3	0	0	—	—	—	—
16	Lung cancer	55	M	24.9	0	0	—	—	—	2	0	0	—	—	—	—
17	Lung cancer	74	F	26.8	0	0	—	—	—	19	0	0	—	—	—	—
18	Benign nodule	60	M	25.3	0	0	—	—	—	2	0	0	—	—	—	—
19	Benign nodule	55	M	28.7	0	0	—	—	—	3	0	0	—	—	—	—
20	Lung cancer	72	F	27.6	0	0	—	—	—	2	0	0	—	—	—	—
21	Benign nodule	57	M	35.1	0	0	—	—	—	4	0	0	—	—	—	—

22	Benign nodule	58	M	21.1	0	0	—	—	—	2	0	0	—	—	—	—
23	Benign nodule	55	M	23.9	0	0	—	—	—	2	0	0	—	—	—	—
24	Benign nodule	58	M	27.3	0	0	—	—	—	2	0	0	—	—	—	—
25	Lung cancer	77	M	20.6	0	0	—	—	—	2	0	0	—	—	—	—
26	Pneumonia	65	M	26.1	1	1	133	Pseudomonas aeruginosa	11	29	1	0	190	35	35	20
27	Pneumonia, AGC	76	M	23.8	1	1	111	Pseudomonas aeruginosa	8	94	0	0	1500	78	7	13
28	Pneumonia, pacreatic cancer	63	M	23.5	1	1	100	Pseudomonas aeruginosa	3	39	1	0	1400	86	5	8
29	Aspiration pneumonia	60	M	16.9	1	1	146	Acinetobacter baumannii	6	125	1	0	175	30	51	19
30	Pneumonia	69	F	22.8	1	1	136	Pseudomonas aeruginosa	1	44	1	0	550	89	11	0
31	Pneumonia, AML	50	M	22.2	1	1	240	—	1	31	1	0	97	90	6	4
32	Pneumonia, thyroid cancer	76	F	28.1	1	1	150	Acinetobacter baumannii	2	34	1	0	284	95	1	2
33	Pneumonia	67	M	17.9	1	1	130	Pseudomonas aeruginosa	4	169	0	0	283	92	8	0
34	Pneumonia	59	M	20.0	1	1	160	Acinetobacter baumannii	9	39	1	0	100	90	6	4
35	Pneumonia	75	M	18.7	1	1	140	Acinetobacter baumannii	9	164	0	0	6800	100	0	0
36	Pneumonia	51	M	23.0	1	1	166	Pseudomonas aeruginosa	2	29	0	0	813	77	21	2
37	Pneumonia	72	M	24.3	1	1	132	Enterobacter cloacae	4	26	1	1	190	63	5	32

38	Pneumonia	73	M	19.2	1	1	123	Acinetobacter baumannii	3	40	1	0	210	76	13	10
39	Pneumonia	69	M	18.9	1	1	142	Acinetobacter baumannii	15	48	1	0	2400	69	25	6
40	Pneumonia	74	M	19.7	1	1	180	Acinetobacter baumannii	6	90	0	0	650	44	3	53
41	Pneumonia	61	M	15.2	1	1	181	Pseudomonas aeruginosa	6	83	0	0	3307	94	1	3
42	Pneumonia	86	M	27.0	1	1	87.5	Klebsiella pneumoniae	2	28	0	0	740	78	5	17
43	Pnuemonia, Lymphoma	78	M	25.4	1	1	140	MRSA	1	37	1	0	1400	97	7	1
44	Pneumonia	84	F	27.3	1	1	240	Acinetobacter baumannii	6	32	1	0	2850	77	17	6
45	Pneumonia	65	M	22.3	1	1	250	Klebsiella pneumoniae	28	73	0	0	3580	90	7	3
46	Aspiration pnuemonia,	60	M	25.3	1	1	160	MRSA	2	46	0	0	300	58	39	3
47	Pneumonia, Esophageal cancer	67	F	24.1	1	1	150	Acinetobacter baumannii	3	31	1	0	660	23	6	32
48	Necrotizing pneumonia, Sigmoid colon cancer	69	M	23.3	1	1	90	Klebsiella pneumoniae	1	2	1	1	8500	98	0	3
49	Pneumonia, Multiple myeloma	75	M	25.7	1	1	117	VRE	1	40	1	0	1586	87	1	12

50	Pneumonia,	62	M	23.1	1	1	250	Klebsiella pneumoniae	2	62	1	0	208	0	28	72
51	Pnuemonia	76	F	22.0	1	1	112	Acinetobacter baumannii	6	15	1	1	850	94	1	5
52	Pneumonia, AML	37	M	21.0	1	1	160	Pseudomonas aeruginosa	0	12	1	1	300	43	21	27
53	Peumonia	77	M	24.9	1	1	150	Acinetobacter baumannii	1	31	1	0	2100	75	8	17
54	Peumonia	80	M	20.2	1	1	240	—	0	8	1	1	26	60	15	10
55	Peumonia	72	M	24.2	1	1	106	Pseudomonas aeruginosa	0	49	1	0	700	23	45	23
56	Peumonia	76	F	27.0	1	1	100	Pseudomonas aeruginosa	0	18	1	1	110	41	29	27
57	Peumonia	59	F	18.1	1	1	220	Enterobacter cloacae	0	36	1	0	1991	95	2	3
58	Peumonia	62	M	24.4	1	1	93	Klebsiella pneumoniae	0	61	0	0	290	76	7	15
59	Peumonia	26	F	19.8	1	1	117	—	0	26	1	1	120	21	53	23
60	Peumonia	45	M	24.1	1	1	237	Acinetobacter baumannii	0	13	1	1	142	84	8	8
61	Peumonia	74	M	24.3	1	1	180	Pseudomonas aeruginosa	1	9	1	1	550	80	8	12

62	Peumonia, Diffuse large B-cell lymphoma	71	F	25.9	1	1	85	Acinetobacter baumannii	0	52	0	0	80	44	47	6
63	Peumonia, Lung cancer	74	M	20.9	1	1	190	Pseudomonas aeruginosa	0	15	1	1	510	56	14	19
64	Pneumonia, Esophageal cancer	64	M	21.2	1	1	242	—	0	18	0	0	694	40	10	41
65	Aspiration pneumonia	26	M	22.4	1	1	260	Pseudomonas aeruginosa	0	29	0	0	Un coun table			
66	Pnuemonia	76	M	22.4	1	1	102	Acinetobacter baumannii	13	80	1	0	Un coun table			

0=no, 1=yes, BMI=body mass index, ARDS=acute respiratory distress syndrome, P/F= PaO_2/FIO_2, BAL=bronchoalveolar lavage, PMN=polymorphonuclear leukocyte, Lympho=lymphocyte, Mono=monocyte, MRSA=Methicillin-resistant Staphylococcus aureus, VRE=vancomycin-resistant Enterococcus.

Sepsis was identified according to the 2001 SCCM/ESICM/ACCP/ATS/SIS International Sepsis Definitions Conference guidelines [4]. Sepsis is defined as SIRS and a known or suspected infection. ARDS was defined according to the Berlin Definition [5]. All ARDS patients were endotracheally intubated and mechanically ventilated using a low tidal volume ventilation strategy according to ARDSNet protocol [6].

Table S3. Primer sequences used for quantitative RT-PCR.

Gene	Primer sequence	PCR product size
Human Pendrin	5′-TTC CCA AAG TGC CAA TCC ATA G-3′<br>5′-CCG CAG TGA TCT CAC TCC AAC-3′	83 bp
Human *Duox2*	5′-ACG CAG CTC TGT GTC AAA GGT-3′<br>5′-TGA TGA ACG AGA CTC GAC AGC-3′	90 bp
Human β-actin	5′-GCA AAG ACC TGT ACG CCA ACA C-3′<br>5′-ATC TCC TTC TGC ATC CTG TC-3′	82 bp
Mouse Pendrin	5’-CAT CTG CAG AAC CAG GTC AA-3’<br>5’-GCA TTC ATC TCT GCC TCC AT-3’	94 bp
Mouse β-actin	5’-TGT TAC CAA CTG GGA CGA CA-3’<br>5’-GGG GTG TTG AAG GTC TCA AA-3’	165 bp

SUPPLEMENTARY REFERENCES

1. el Marjou F, Janssen KP, Chang BH, *et al.* Tissue-specific and inducible Cre-mediated recombination in the gut epithelium. Genesis. 2004; 39:186-193.

2. Gui YS, Wang L, Tian X, *et al.* SPC-Cre-ERT2 transgenic mouse for temporal gene deletion in alveolar epithelial cells. PLoS One. 2012; 7:e46076.

3. Matute-Bello G, Downey G, Moore BB, *et al.* An official American Thoracic Society workshop report: features and measurements of experimental acute lung injury in animals. Am J Respir Cell Mol Biol. 2011; 44:725-738.

4. Levy MM, Fink MP, Marshall JC, *et al.* 2001 SCCM/ESICM/ACCP/ATS/SIS International Sepsis Definitions Conference. Crit Care Med. 2003; 31:1250-1256.

5. Ranieri VM, Rubenfeld GD, Thompson BT, *et al.* Acute respiratory distress syndrome: the Berlin Definition. Jama. 2012; 307:2526-2533.

6. Brower RG, Matthay MA, Morris A, Schoenfeld D, Thompson BT, Wheeler A. Ventilation with lower tidal volumes as compared with traditional tidal volumes for acute lung injury and the acute respiratory distress syndrome. N Engl J Med. 2000; 342:1301-1308.
